# A long-term high-fat diet induces differential gene expression changes in spatially distinct adipose tissue of male mice

**DOI:** 10.1152/physiolgenomics.00080.2024

**Published:** 2024-09-30

**Authors:** Malak Alradi, Hassan Askari, Mark Shaw, Jaysheel D. Bhavsar, Brewster F. Kingham, Shawn W. Polson, Ibra S. Fancher

**Affiliations:** ^1^Department of Biological Sciences, College of Arts and Sciences, University of Delaware, Newark, Delaware, United States; ^2^Department of Medical Biotechnology, School of Advanced Medical Sciences and Technologies, Shiraz University of Medical Sciences, Shiraz, Iran; ^3^Delware Biotechnology Institute, University of Delaware, Newark, Delaware, United States; ^4^Center for Bioinformatics and Computational Biology, University of Delaware, Newark, Delaware, United States; ^5^Department of Computer and Information Sciences, College of Engineering, University of Delaware, Newark, Delaware, United States; ^6^Department of Kinesiology and Applied Physiology, College of Health Sciences, University of Delaware, Newark, Delaware, United States

**Keywords:** adipose tissue, obesity, RNA sequencing, subcutaneous adipose, visceral adipose

## Abstract

The accumulation of visceral adipose tissue (VAT) is strongly associated with cardiovascular disease and diabetes. In contrast, individuals with increased subcutaneous adipose tissue (SAT) without corresponding increases in VAT are associated with a metabolic healthy obese phenotype. These observations implicate dysfunctional VAT as a driver of disease processes, warranting investigation into obesity-induced alterations of distinct adipose depots. To determine the effects of obesity on adipose gene expression, male mice (*n* = 4) were fed a high-fat diet to induce obesity or a normal laboratory diet (lean controls) for 12–14 mo. Mesenteric VAT and inguinal SAT were isolated for bulk RNA sequencing. AT from lean controls served as a reference to obesity-induced changes. The long-term high-fat diet induced the expression of 169 and 814 unique genes in SAT and VAT, respectively. SAT from obese mice exhibited 308 differentially expressed genes (164 upregulated and 144 downregulated). VAT from obese mice exhibited 690 differentially expressed genes (262 genes upregulated and 428 downregulated). KEGG pathway and GO analyses revealed that metabolic pathways were upregulated in SAT versus downregulated in VAT while inflammatory signaling was upregulated in VAT. We next determined common genes that were differentially regulated between SAT and VAT in response to obesity and identified four genes that exhibited this profile: *elovl6* and *kcnj15* were upregulated in SAT/downregulated in VAT while *trdn* and *hspb7* were downregulated in SAT/upregulated in VAT. We propose that these genes in particular should be further pursued to determine their roles in SAT versus VAT with respect to obesity.

**NEW & NOTEWORTHY** A long-term high-fat diet induced the expression of more than 980 unique genes across subcutaneous adipose tissue (SAT) and visceral adipose tissue (VAT). The high-fat diet also induced the differential expression of nearly 1,000 AT genes. We identified four genes that were oppositely expressed in SAT versus VAT in response to the high-fat diet and propose that these genes in particular may serve as promising targets aimed at resolving VAT dysfunction in obesity.

## Graphical abstract

**Figure:**
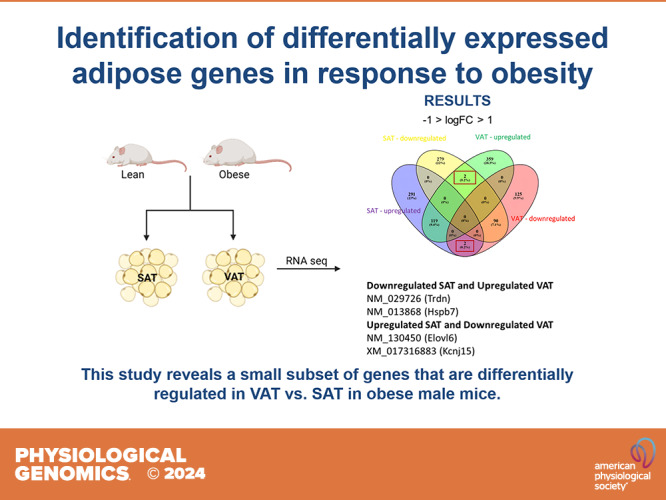

## INTRODUCTION

Adipose tissue (AT) is now recognized as much more than a storage site for lipids and its role as an endocrine/paracrine tissue is now well-accepted. Although this clearly affords AT to play major roles in systems physiology, it also implicates AT dysfunction as a potential central piece in the development of metabolic disorders. As such, visceral obesity, the accumulation of abdominal intraperitoneal adipose tissue, is strongly associated with the development of diabetes and cardiovascular disease (1–4). The metabolic unhealthy obese phenotype, accompanied by chronic inflammation, insulin resistance, and adipose tissue (AT) dysfunction, is often observed in individuals with elevated visceral adipose tissue (VAT). In contrast, those that possess increased subcutaneous adipose tissue (SAT) without corresponding increases in VAT denote the metabolic healthy obese phenotype and are not associated with similar inflammatory and metabolic deficits (1, 5–7).

These observations point to a differential role for spatially distinct AT in potentially underlying these metabolic phenotypes and warrant an investigation into the relative AT gene expression alterations that occur in obesity, particularly with the accumulation of VAT. In doing so, we may reveal novel targets and pathways that may better prevent obesity-associated diseases in those afflicted with VAT dysfunction. Here, we profiled and analyzed gene expression in SAT and VAT from obese C57BL/6J mice on a long-term (i.e., 12–14 mo) high-fat diet relative to lean control counterparts maintained on a normal laboratory diet to identify differentially expressed genes (DEGs) in these distinct ATs via bulk RNA sequencing. We identified significant DEGs and associated pathways and assessed gene ontology. Our major goal was to identify genes that were up/downregulated in opposite directions in SAT versus VAT with obesity relative to SAT and VAT from lean mice as we propose that these genes in particular may offer future therapeutic potential.

## MATERIALS AND METHODS

### Animals and Diets

All animal experiments were approved by the Institutional Animal Care and Use Committees at the University of Delaware. Eight male C57BL/6J mice at 10 wk old were divided into two diet groups: an age**-**matched lean control group maintained on a normal laboratory rodent diet and an obese group fed a high-fat Western diet. The high-fat diet (purchased from Inotiv; Cat. No. TD.88137) contains (by weight) 17.3% protein, 48.5% carbohydrates, 21.2% fat, and 0.2% cholesterol. This corresponds to 15.2%, 42.7%, and 42% kcal, respectively, from the macronutrients present in the high-fat diet. In contrast, the standard rodent diet (Lab Diet, Prolab RMH 3000; Cat. No. 5P00) contains 26.1% kcal from protein, 14.4% kcal from fat, 59.5% kcal from carbohydrates, and 0.02% cholesterol. Respective diets, beginning at 10 wk old, and water were given ad libitum. The corresponding diets were maintained for 12–14 mo. Mice were euthanized between 62 and 70 wk of age. The mice were housed at the University of Delaware Life Sciences Research Facility animal vivarium under standard 12-h light and dark cycles. Mice were euthanized using CO_2_ asphyxiation followed by cervical dislocation.

### Adipose Isolation, Total RNA Extraction, and RNA-Seq Library Preparation

Inguinal SAT and mesenteric VAT were isolated from four lean and four obese mice generating 16 adipose samples (i.e., 8 SAT and 8 VAT samples total) for bulk RNA-seq processing and analysis. Both left and right inguinal SAT depots were isolated and combined to generate a single sample of SAT per mouse. Visible vasculature was manually dissected and removed from AT, which was stored in liquid nitrogen until all samples were ready for downstream processing and RNA sequencing. Adipose tissue samples were submitted to the UD Sequencing and Genotyping Center (RRID:SCR_012230) for RNA sequencing. Total RNA extraction was performed using the Qiagen RNeasy Universal kit (Qiagen, Germantown, MD) as per manufacturer’s guidelines. Quality analysis was performed and high-quality RNA was verified using a Fragment Analyzer 5200 (Agilent, Santa Clara, CA). Poly-A RNAseq library preparation was done using Perkin Elmer NEXTFLEX Rapid Directional RNAseq 2.0 and NEXTFLEX Poly(A) Beads 2.0 library preparation kits (Perkin Elmer, Waltham, MA) as per manufacturer’s guidelines using 1 μg of total RNA as library preparation input.

### RNA-Seq Analysis

mRNA was sequenced on an Illumina NextSeq 2000 (Illumina, San Diego, CA) producing paired**-**end 101 bp sequence reads. Sequence data were analyzed by the University of Delaware Bioinformatics Data Science Core Facility (RRID:SCR_017696) using a customized version of the MAPRseq pipeline (8). Sequence reads were assessed for quality using FastQC (v0.11.9; Babraham Bioinformatics) and trimmed using TrimGalore! (v0.11.9) to remove illumina sequencing adapters, poly-G sequence at read ends, and sequences not meeting a quality threshold of Q30. After trimming, reads that did not meet the desired threshold of 75 bp were discarded. Accepted reads were aligned to the mouse genome (GRCm39 refseq Annotation 109) using HiSat2 (v2.2.0) (9), mapping quality was assessed using RseQC (v4.0.0) (10), and counts for gene features were determined using HTseq (v0.13.5) (11) Features not occurring at CPM >1 in three samples were removed and differential gene expression analysis was performed with EdgeR (v3.34.0) (12) using generalized linear models to assess contrasts while controlling for paired samples from the same individual. Significantly differentially expressed features were determined using the Quasi-likelihood F test with FDR correction (q < 0.05). The Venn diagram for detection of unique gene expression was generated using Venny 2.1 software and is represented as a percentage relative to the total percent of expressed genes (CPM > 1).

### Differential Expression Gene Analyses

Pairwise differential expression analyses were run using EdgeR for the following diet treatment and adipose depot comparisons: SAT from obese mice relative to SAT from lean mice and VAT from obese mice relative to VAT from lean mice. Upregulated and downregulated genes in AT are denoted as positive or negative base 2 logarithmic fold change (logFC) values relative to gene expression of AT from lean control counterparts. A gene was considered to be differentially expressed using an adjusted false discovery rate (FDR) < 0.05 and an absolute fold change of −1.5 > logFC > 1.5. Volcano plots were generated using Python, and the heatmaps generated using R. Venn diagrams were generated using Venny 2.1 software. Unknown genes were not included for representation in the present study.

### KEGG Pathway, Gene Ontology, and Pathway-Pathway Network Analyses

Pathways analysis was performed using the Kyoto Encyclopedia of Genes and Genomes (KEGG) database (https://www.kegg.jp/), as described previously (13, 14), using DEG with an adjusted FDR < 0.05, and −1.5 > logFC > 1.5. Gene ontology (GO) is a system of functional classification of genes that includes the biological process (BP), cellular component (CC), and molecular function (MF). GO analysis was performed using The Database for Annotation, Visualization and Integrated Discovery (DAVID) (15) with FDR < 0.05 using DEG with an adjusted FDR < 0.05, and −1.5 > logFC >1.5. Pathway‐pathway network analyses were generated using the ClueGo plugin within Cytoscape (16) with *P* value set to < 0.05. The pathway-pathway networks were based on enriched KEGG pathways and GO terms from the identified DEGs in SAT and VAT following the long-term high-fat diet.

## RESULTS

### Long-Term High-Fat Diet Induces Expression of Unique Genes in SAT and VAT

**Figure 1.**
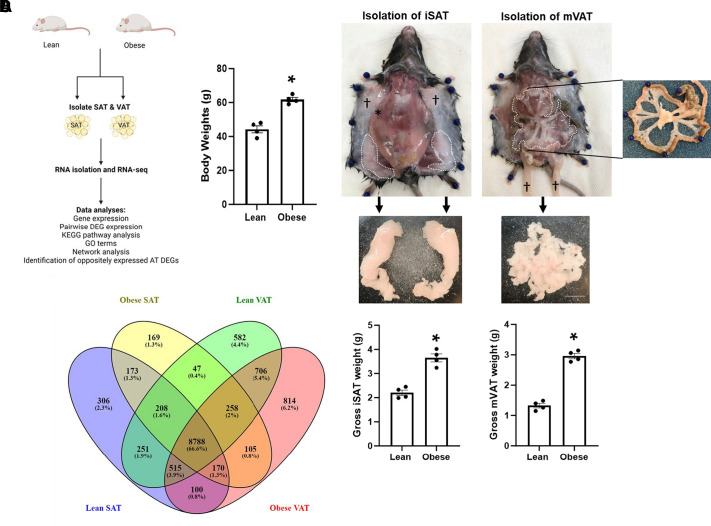
SAT and VAT gene expression in mice following a long-term high-fat diet versus a normal laboratory diet as assessed by RNA sequencing. *A*: schematic of experimental design for RNA sequencing and data analyses using SAT and VAT from lean and obese mice. Following 12–14 mo on respective diets, obesity-induced changes in AT gene expression were determined by assessing pairwise fold changes in gene expression relative to lean AT gene expression. KEGG pathway and GO term analyses, and the identification of DEGs with opposite AT expression patterns, followed in the same format. The schematic was generated using BioRender. *B*: body weights of lean and obese mice at the conclusion of the diet regimen (*n* = 4 mice/group; *P < 0.001 using a one-tailed Student’s *t* test). *C*: representation of the dissection method used to target inguinal SAT (iSAT) and mesenteric VAT (mVAT) depots in the present study. The targeted AT depots are outlined with a white dashed line (*top*). †AT not used in the present study. Note: the inset depicting the isolated bowel is to further highlight the targeted VAT depot. mVAT was isolated directly from the intact bowel for these studies. Also shown are images of the isolated iSAT and mVAT (*middle*) and corresponding gross weights (g) of isolated SAT and VAT from lean and obese mice (*bottom*; *n* = 4 mice/group; *P < 0.001 and 0.00001, respectively using a one-tailed Student’s *t* test). Scale bar is 5 mm. Note: left and right isolated iSAT depots from each mouse were weighed together and represent a single data point per mouse. *D*: Venn diagram showing a comparison of expressed genes from SAT and VAT of lean and obese mice (*n* = 4 mice/group). The number of expressed genes that are common or unique to a specific AT are represented by a percentage relative to the total number of detected genes. AT, adipose tissue; DEGs, differentially expressed genes; SAT, subcutaneous adipose tissue; VAT, visceral adipose tissue.

Male C57BL/6J mice were fed either a high-fat diet to induce obesity or a normal laboratory diet (lean controls) for 12**–**14 mo before the isolation of SAT and VAT. A schematic detailing the general workflow of AT isolation before RNA sequencing and analysis is shown in Fig. 1*A*. As expected, mice fed the high-fat diet weighed significantly more than lean controls at the conclusion of the long-term diet regimen (44.3 ± 2.1 g vs. 61.8 ± 1.3 g; *P* < 0.001), exhibiting an average ∼40% increase in body weight (Fig. 1*B*). We targeted specific SAT and VAT depots for comparison in this study with each individual mouse providing two inguinal SAT depots and a mesenteric VAT depot for analysis. The top panel of Fig. 1*C* denotes the respective targeted depots in a representative dissection of a 14-mo-old mouse fed the standard laboratory diet. The two inguinal SAT depots are depicted in dashed outlines in the dissected mouse with images of the isolated SAT tissues shown below (Fig. 1*C*, *top* and *middle*, respectively). We did not isolate and collect the upper, pectoral SAT (denoted by † in the image) for these studies. We similarly depict the targeted mesenteric VAT depot in the same representative dissection and further show an inset of isolated bowel to better reveal the VAT of interest. It is important to note that this particular representation is for clarity only and VAT is isolated from the intact bowel to avoid bacterial contamination that would occur with complete removal of the gut. The isolated mesenteric VAT is depicted below the representative dissection image. We did not use epididymal VAT (denoted by † in the dissection image) in this study. As expected, both AT depots isolated from the mice fed the high-fat diet were significantly greater in mass compared with respective AT from lean controls (Fig. 1*C*, *bottom*). Isolated inguinal SAT and mesenteric VAT from lean and obese mice were then processed for total RNA that was then subjected to RNA sequencing and analysis. We detected a total of 13,192 AT genes in samples from the eight male mice with 8,788 (66.6%) of the genes being commonly expressed in all four groups (i.e., SAT and VAT from lean or obese mice; Fig. 1*D*). In AT isolated from lean mice, SAT exhibited an expression of 306 (2.3%) unique genes while VAT expressed 582 (4.4%) unique genes. The high-fat diet induced the expression of 169 (1.3%) unique genes in SAT, whereas a remarkable 814 (6.2%) unique genes were induced in VAT (Fig. 1*B*). The high-fat diet also induced the expression of 105 (0.8%) genes in both SAT and VAT that were not detected in AT of lean controls. These data suggest that SAT and VAT undergo differential gene expression remodeling in response to long-term high-fat diet.

### Long-Term High-Fat Diet Induces Differential Gene Expression in SAT Versus VAT

**Figure 2.**
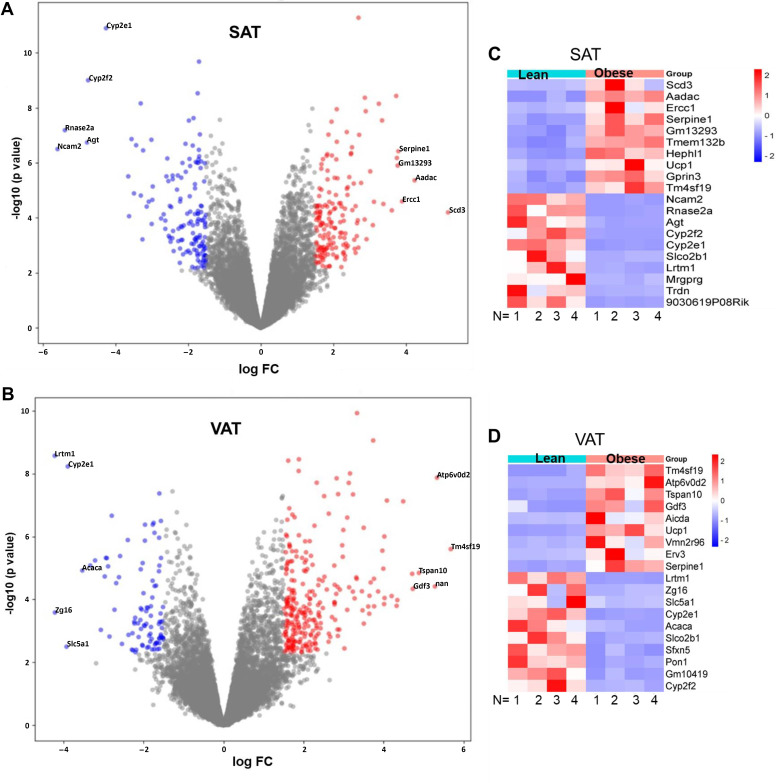
A long-term high-fat diet induces differential gene expression in SAT versus VAT. Volcano plots reveal changes in gene expression with obesity in SAT (*A*) and VAT (*B*). Obesity-induced alterations in gene AT expression are denoted as the log fold change (FC) relative to AT from lean mice (*n* = 4 mice/group). Red denotes significantly upregulated genes, whereas blue denotes significantly downregulated genes. The top five up- and downregulated genes are labeled in the plots in both *A* and *B*. Heat maps reveal the top 20 genes significantly up- or downregulated in SAT (*C*) and VAT (*D*) relative to an average CPM for all 8 (i.e., 4 lean, 4 obese) SAT or VAT samples for a given gene with the logCPM for each gene being normalized to library size. Red indicates scaled levels of upregulation and blue indicates scaled levels of downregulation. For a gene to be considered significant, FDR < 0.5 and −1.5 > logFC > 1.5. AT, adipose tissue; SAT, subcutaneous adipose tissue; VAT, visceral adipose tissue.

We next determined AT genes that were differentially up- or downregulated in response to the long-term high-fat diet. Volcano plots reveal that relative to lean control mice, 308 genes were differentially expressed in SAT from obese mice (Fig. 2*A*). This includes 164 significantly upregulated genes and 144 significantly downregulated genes (FDR < 0.05. −1.5> logFC >1.5). In contrast, 690 genes were differentially expressed in VAT from obese mice with 262 significantly upregulated genes and 428 significantly downregulated genes (FDR < 0.05. −1.5> logFC >1.5) (Fig. 2*B*). Of these differentially expressed genes (DEGs), the top 20 significantly up- and downregulated genes with the greatest fold change are shown in the heat maps for each AT type (Fig. 2, *C* and *D*). The functions of these specific DEGs are shown in Tables 1 (SAT DEGs) and 2 (VAT DEGs).

**Table 1.** The top 20 significant DEGs in SAT with the Gene ID, gene name, log_2_ fold change with obesity compared with lean (−1.5 > logFC > 1.5), FDR P value (P < 0.05), and gene function

	Gene ID	Gene Name	log_2_ (Fold Change)	FDR *P* Value	Gene Function
SAT	ENSMUSG00000025202	*scd3*	5.16	0.0022	Enables palmitoyl-CoA 9-desaturase activity and stearoyl-CoA 9-desaturase activity.
	ENSMUSG00000027761	*aadac*	4.24	0.0004	Enables serine hydrolase activity and triglyceride lipase activity
	ENSMUSG00000003549	*ercc1*	3.90	0.0013	Enables TFIID-class transcription factor complex binding activity and promoter-specific chromatin binding activity
	ENSMUSG00000037411	*serpine1*	3.80	0.0001	Enables serine-type endopeptidase inhibitor activity
	ENSMUSG00000086006	*gm13293*	3.77	0.0002	ncRNA
	ENSMUSG00000070498	*tmem132b*	3.75	0.0001	Transmembrane protein
	ENSMUSG00000031936	*hephl1*	3.74	6.87E-06	Predicted to enable ferroxidase activity
	ENSMUSG00000031710	*ucp1*	3.61	0.0020	Enables long-chain fatty acid binding activity
	ENSMUSG00000045441	*gprin3*	3.38	0.0015	Predicted to be involved in neuron projection development
	ENSMUSG00000079625	*tm4sf19*	3.12	0.0013	Predicted to be integral component of membrane
	ENSMUSG00000022762	*ncam2*	−5.59	9.77E-05	Predicted to enable identical protein binding activity. Predicted to be involved in cell adhesion
	ENSMUSG00000047222	*rnase2a*	−5.39	3.82E-05	Predicted to enable ribonuclease activity
	ENSMUSG00000031980	*agt*	−4.78	7.00E-05	Enables type 1 angiotensin receptor binding activity and type 2 angiotensin receptor binding activity
	ENSMUSG00000052974	*cyp2f2*	−4.75	2.77E-06	Predicted to enable heme binding activity and monooxygenase activity
	ENSMUSG00000025479	*cyp2e1*	−4.26	7.09E-08	Enables monooxygenase activity
	ENSMUSG00000030737	*slco2b1*	−3.65	0.0003	Predicted to enable bile acid transmembrane transporter activity and sodium-independent organic anion transmembrane transporter activity
	ENSMUSG00000045776	*lrtm1*	−3.56	5.90E-05	Predicted to enable roundabout binding activity and heparin binding activity
	ENSMUSG00000050276	*mrgprg*	−3.49	0.0009	Predicted to enable G protein-coupled receptor activity
	ENSMUSG00000019787	*trdn*	−3.43	7.96E-05	Predicted to enable transmembrane transporter binding activity.
	ENSMUSG00000053168	*9030619P08rik*	−3.30	8.82E-06	RIKEN cDNA 9030619P08 gene

DEGs, differentially expressed genes; SAT, subcutaneous adipose tissue.

**Table 2.** The top 20 significant DEGs in VAT with the Gene ID, gene name, log_2_ fold change with obesity compared with lean (−1.5 > logFC > 1.5), FDR P value (P < 0.05), and gene function

	Gene ID	Gene Name	log_2_ (Fold Change)	FDR *P* Value	Gene Function
VAT	ENSMUSG00000079625	*tm4sf19*	5.68	0.0004	Predicted to be integral component of membrane
	ENSMUSG00000028238	*atp6v0d2*	5.33	1.69E-05	Predicted to enable proton transmembrane transporter activity
	ENSMUSG00000039691	*tspan10*	4.88	0.0012	Enables enzyme binding activity
	ENSMUSG00000030117	*gdf3*	4.73	0.0025	Secreted ligand of the TGF-β (transforming growth factor-beta) superfamily of proteins
	ENSMUSG00000040627	*aicda*	4.71	0.0012	Enables cytidine deaminase activity
	ENSMUSG00000031710	*ucp1*	4.49	4.16E-05	Enables long-chain fatty acid binding activity
	ENSMUSG00000091679	*vmn2r96*	4.34	0.0043	Predicted to enable G protein-coupled receptor activity
	ENSMUSG00000037482	*erv3*	4.32	0.0055	NA
	ENSMUSG00000079625	*tm4sf19*	4.20	0.0035	Predicted to be integral component of membrane
	ENSMUSG00000037411	*serpine1*	4.10	4.10E-05	Enables serine-type endopeptidase inhibitor activity.
	ENSMUSG00000045776	*lrtm1*	−4.22	9.27E-06	Predicted to enable roundabout binding activity and heparin binding activity
	ENSMUSG00000049350	*zg16*	−4.22	0.0074	Enables peptidoglycan binding activity
	ENSMUSG00000011034	*slc5a1*	−3.93	0.0374	Enables glucose transmembrane transporter activity
	ENSMUSG00000025479	*cyp2e1*	−3.89	1.18E-05	Enables monooxygenase activity
	ENSMUSG00000020532	*acaca*	−3.53	0.0010	Enables acetyl-CoA carboxylase activity
	ENSMUSG00000030737	*slco2b1*	−3.21	0.0006	Predicted to enable bile acid transmembrane transporter activity and sodium-independent organic anion transmembrane transporter activity
	ENSMUSG00000033720	*sfxn5*	−3.07	0.0169	Predicted to enable citrate transmembrane transporter activity
	ENSMUSG00000002588	*pon1*	−2.97	0.0014	Enables arylesterase activity
	ENSMUSG00000072769	*gm10419*	−2.94	0.0006	NA
	ENSMUSG00000052974	*cyp2f2*	−2.92	0.0006	Predicted to enable heme binding activity and monooxygenase activity

DEGs, differentially expressed genes; VAT, visceral adipose tissue.

### Long-Term High-Fat Diet Induces Stark Differences in AT DEG-Related Pathways

**Figure 3.**
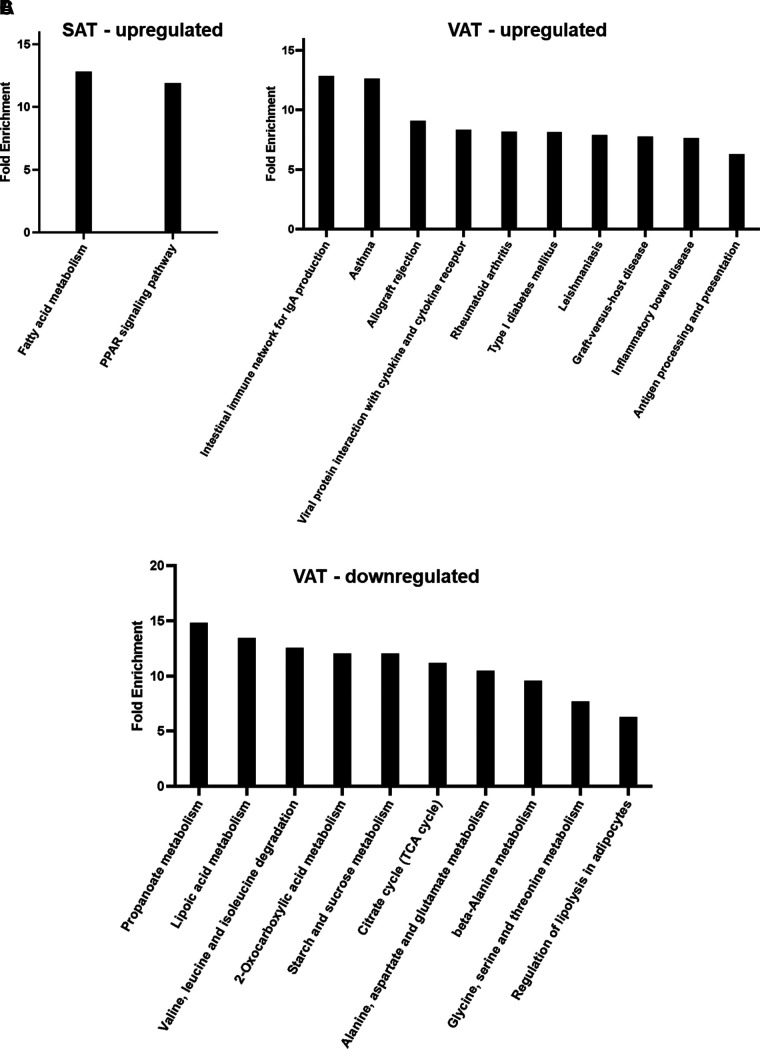
A long-term high-fat diet influences metabolic and inflammatory signaling in SAT and VAT. KEGG pathway enrichment analysis shows the influenced signaling and metabolic pathways, as well as associated diseases, with which the significantly expressed DEGs in SAT (*A*) and VAT (*B* and *C*) are associated (*n* = 4 mice/group). *A*: two upregulated pathways were identified in SAT, whereas 44 pathways were identified in VAT. The top 10 upregulated (*B*) and downregulated (*C*) pathways are shown in VAT with obesity. Fold enrichment represents the percent of the genes from the RNA-sequencing data that belong to the pathway (FDR < 0.05). DEGs, differentially expressed genes; KEGG, Kyoto Encyclopedia of Genes and Genomes; SAT, subcutaneous adipose tissue; VAT, visceral adipose tissue.

KEGG pathway analysis offers a connection between genomic information with functional information that is represented in a pathway (13, 14). The identified DEGs were significantly enriched in 46 KEGG pathways (FDR < 0.05) across SAT and VAT of obese mice relative to lean controls. Only two pathways were enriched in SAT as compared with 44 enriched pathways in VAT (Fig. 3*A*, Supplemental Table S1). The upregulated pathways in SAT were Fatty acid metabolism and the PPAR signaling pathway, each with fold enrichments >10 (Fig. 3*A*). There were no significantly downregulated pathways identified in SAT of obese mice. The upregulated DEG in VAT were enriched in 24 pathways with a majority being related to inflammation (the top 10 pathways with the greatest fold change are shown in Fig. 3*B*). Of these, the intestinal immune network for IgA production and asthma were the most influenced pathways, each with fold enrichments >10. The downregulated VAT DEGs were enriched in 20 pathways largely related to metabolism (the top 10 pathways with the greatest fold change are shown in Fig. 3*C*, the most significantly influenced pathway being propanoate metabolism, which approaches a fold enrichment of 15). The remaining significantly enriched pathways are detailed in the online Supplemental Table S1.

### Long-Term High-Fat Diet Induces Stark Differences in AT Biological Processes and Molecular Functions

We next used Gene Ontology (GO) as a system to functionally classify the identified DEGs in SAT and VAT. This provides a more complete picture of the changing DEGs as they relate to specific biological processes (BPs), cellular components (CCs), and molecular functions (MFs) that may encompass several altered DEGs. Specific GO terms within each major category were detected using the DAVID database (15). The DEGs were significantly enriched in 109 GO terms (FDR < 0.05) spanning the three GO categories in SAT and VAT with obesity (Supplemental Tables S2 and S3). There were far fewer GO terms that were enriched in SAT (Supplemental Table S2) than in VAT (Supplemental Table S3). Similar to the KEGG pathway analysis, we did not identify significantly downregulated GO terms in SAT of obese mice with an FDR < 0.05. These findings were further supported when using the KEGG pathways and GO terms in pathway-pathway network analyses generated using ClueGo functionality in Cytoscape (16). This analysis revealed a remarkable difference in the number of interrelated pathways influenced in response to obesity with VAT exhibiting a pronounced increase in the number of networks as compared with SAT (Supplemental Fig. S1).

**Figure 4.**
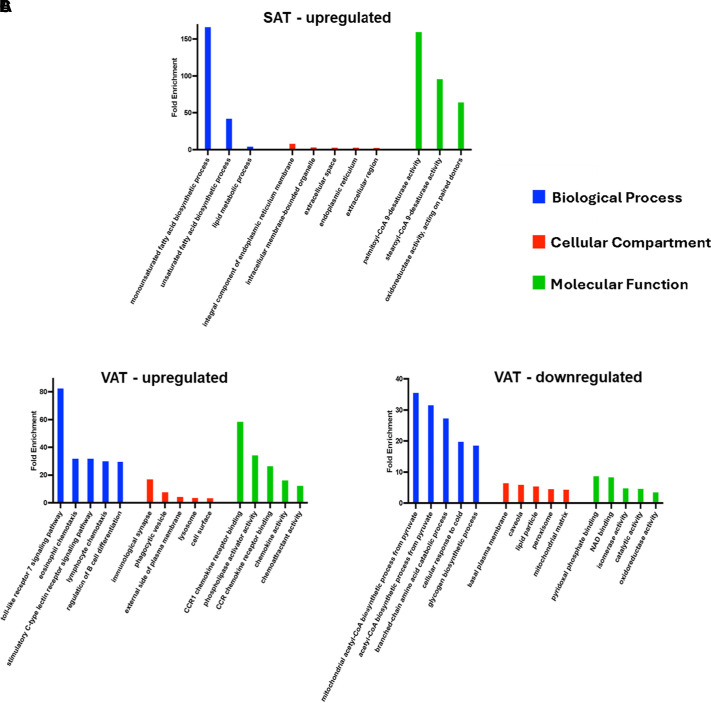
A long-term high-fat diet influences biological processes and molecular functions associated with metabolism and inflammation in SAT and VAT. GO terms incorporating biological processes (BPs), cellular components (CCs), and molecular functions (MFs) of the top upregulated DEGs in SAT (*A*) and the top five GO terms for each category related to the upregulated (*B*) and downregulated (*C*) DEGs in VAT (FDR < 0.05) with obesity (*n* = 4 mice/group). The full list of up- and downregulated GO terms associated with VAT can be found in the online supplement. DEGs, differentially expressed genes; SAT, subcutaneous adipose tissue; VAT, visceral adipose tissue.

Similar to the KEGG pathway analysis, the upregulated DEGs in SAT pertaining to BP were related to fatty acid biosynthetic processes and lipid metabolism, the most influenced of which was monounsaturated fatty acid biosynthetic process with a fold enrichment >150; the upregulated DEGs in SAT of the CC were enriched in the ER membrane with the most affected compartment being integral component of endoplasmic reticulum membrane; and the upregulated SAT DEGs related to MF were mainly involved with fatty acid desaturase activity with palmitoyl-CoA 9-desaturase activity having a fold enrichment >150 (Fig. 4*A*). The full list of significantly enriched GO terms relevant to SAT can be found in the online supplement (Supplemental Table S2). Also similar to the KEGG pathways observed in VAT following the long-term high**-**fat diet, the VAT DEGs that were upregulated in BP were mainly related to inflammation (Toll-like receptor 7 signaling pathway exhibited a fold enrichment >80); the upregulated VAT DEGs of the CC were highly enriched in the immunological synapse with fold enrichment approaching 20; and the upregulated VAT DEGs of MF were mainly involved in CCR1 chemokine receptor binding with fold enrichment approaching 60 (Fig. 4*B*). Furthermore, the downregulated VAT DEGs of BP were mainly involved in metabolic processes (both mitochondrial acetyl-CoA biosynthetic process from pyruvate and acetyl-CoA biosynthetic process from pyruvate exhibited fold enrichments >30); the downregulated VAT DEGs of the CC were mainly associated with the plasma membrane, caveola, and lipid particles; and the downregulated VAT DEGs of MF were mainly involved in pyridoxal phosphate binding and NAD binding, each approaching fold enrichments of 10 (Fig. 4*C*). The full list of significantly enriched GO terms relevant to VAT can be found in the online supplement (Supplemental Table S3).

### Long-Term High-Fat Diet Induces Differential Expression of a Select Subset of AT Genes

**Figure 5.**
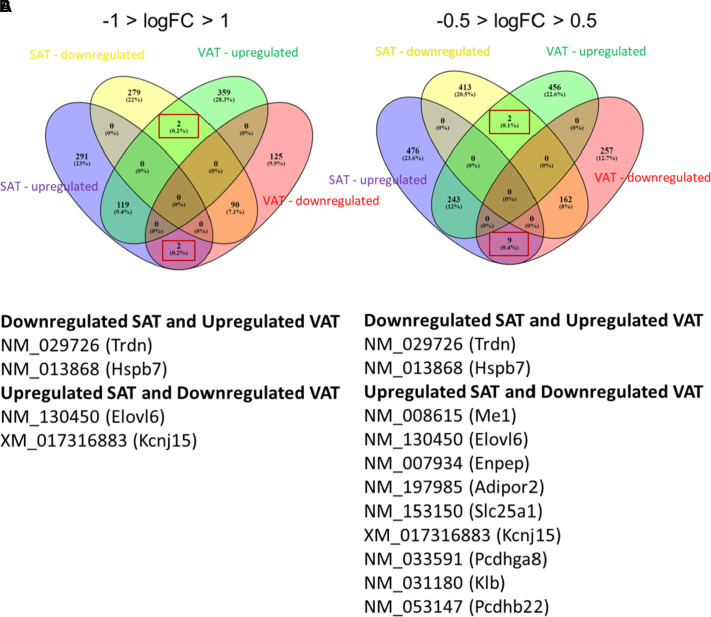
A long-term high-fat diet induces differential expression of four common genes in SAT versus VAT. Venn diagrams highlight the low number of genes that are oppositely expressed in SAT versus VAT in response to the long-term high-fat diet using fold change (FC) stringency levels of −1 > logFC > 1 (*A*) and −0.5 > logFC > 0.5 (*B*). The boxes in each diagram reveal the overlapping genes with differential expression patterns and the tables reveal that we detected 4 (*A*) versus 11 (*B*) specific DEGs that were expressed in this fashion (*n* = 4 mice/group). Each gene name in the table is accompanied by its gene reference number. DEGs, differentially expressed genes; SAT, subcutaneous adipose tissue; VAT, visceral adipose tissue.

The next goal was to identify genes upregulated in SAT and downregulated in VAT, and vice versa, following the long-term high**-**fat diet. Based on the pathological status associated with VAT as opposed to SAT with obesity (2–4, 6, 7), we propose that identifying such differentially expressed AT genes may offer promising therapeutic avenues. Surprisingly, only four total genes exhibited such a profile with *trdn* and *hspb7* downregulated in SAT/upregulated in VAT and *elvol6* and *kcnj15* upregulated in SAT/downregulated in VAT in response to obesity and relative to lean controls (Fig. 5). When the stringency of the logFC parameters was weakened from −1 > logFC > 1 to −0.5 > logFC > 0.5, this modestly increased the number of AT DEGs significantly influenced in opposite directions to 11, and all seven of the additional genes were upregulated in SAT/downregulated in VAT (Fig. 5 and Table 3). Table 3 also details the FDR *P* value, fold change, and function of each DEG oppositely expressed in SAT versus VAT in response to the long-term high-fat diet.

**Table 3.** The 11 genes that are oppositely expressed in SAT and VAT with obesity using a fold change threshold of β0.5 > logFC > 0.5; FDR < 0.05

Gene ID	Gene Name	log2 (fold change)	FDR *P* Value	log2 (fold change)	FDR *P* value	Gene Function
ENSMUSG00000019787	*trdn	−3.43	7.96E-05	2.07	0.0060	Predicted to enable transmembrane transporter binding activity
ENSMUSG00000006221	*hspb7	−2.22	0.0013	1.42	0.0034	Enables filamin binding activity.
ENSMUSG00000032418	*me1*	1.37	3.94E-05	−0.88	0.0287	Enables malate dehydrogenase (decarboxylating) (NADP+) activity.
ENSMUSG00000041220	*elovl6	1.06	0.0013	−1.19	0.0012	Enables acyltransferase activity
ENSMUSG00000028024	*enpep*	0.58	0.0021	−1.61	3.43E-05	Enables peptidase activity
ENSMUSG00000030168	*adipor2*	0.62	0.0043	−0.74	0.0186	Enables adiponectin binding activity; identical protein binding activity; and signaling receptor activity.
ENSMUSG00000003528	*slc25a1*	0.54	0.0088	−0.78	0.0012	Predicted to enable citrate secondary active transmembrane transporter activity
ENSMUSG00000062609	*kcnj15	1.00	0.0203	−1.34	0.0463	Enables potassium channel activity.
ENSMUSG00000103897	*pcdhga8*	0.94	0.0207	−0.92	0.0463	NA
ENSMUSG00000029195	*klb*	0.54	0.0219	−0.85	0.0312	Predicted to enable fibroblast growth factor binding activity and fibroblast growth factor receptor binding activity.
ENSMUSG00000073591	*pcdhb22*	>0.50	0.0226	−0.74	0.0213	Predicted to be involved in cell adhesion.
		SAT		VAT		

SAT, subcutaneous adipose tissue; VAT, visceral adipose tissue. *Denotes one of four genes that remains significant after the more stringent threshold of −1 > logFC > 1.

## DISCUSSION

AT plays a multifactorial role in metabolic and inflammatory status and AT dysfunction is now predicted to be a culprit in the progression of cardiovascular disease and diabetes. VAT in particular has been a major focal point to date where, in contrast to SAT, VAT accumulation is strongly associated with increased macrophage infiltration and inflammation, increased adipocyte size, and decreased lipid metabolism (17–22). Therefore, unveiling the gross transcriptome changes that differ between SAT and VAT under obesogenic conditions may lead to a better understanding of VAT dysfunction and offer an initial platform of potential targets aimed at preventing or even reversing specific AT depot dysfunction.

In this study, male C57Bl/6J mice were fed a high-fat diet for 12–14 mo, which resulted in expected increases in body weight as well as SAT and VAT mass. Although the SAT from obese mice exhibited a 65.6% increase in mass compared with SAT from lean controls, VAT showed a stark 123.2% increase in mass relative to VAT from lean mice, immediately revealing differences in the response to the high-fat diet between these AT depots. Our major goal in this study was to examine the gene expression profiles via RNA sequencing of SAT and VAT to identify AT-specific differences in response to obesity. Changes in gene expression in response to the high-fat diet were assessed relative to lean control mice maintained on a normal laboratory diet. Our initial assessment identified more than 13,000 AT genes with 1.3% and 6.2% being uniquely expressed in SAT and VAT, respectively, in response to the long-term high-fat diet. A total of 308 DEGs were identified in SAT, with 164 genes being upregulated versus 144 downregulated while nearly 700 DEGs were identified in VAT with 262 being upregulated versus 428 downregulated as compared with matched lean control AT. The DEGs identified in SAT and VAT were significantly enriched in several pathways related to metabolism (e.g., fatty acid metabolism, amino acid metabolism, carbohydrate metabolism, TCA cycle) and inflammation (e.g., IgA production, cytokines and cytokine receptors, and antigen processing/presentation). Interestingly, SAT DEGs were only enriched in two upregulated pathways driving lipid metabolism and were not significantly associated with downregulated pathways. The upregulated DEGs in SAT support a role for enhanced metabolic activity related to lipid handling in response to nutrient overload. In contrast, VAT from obese mice was significantly associated with a total of 44 differentially regulated pathways. Of note, in the upregulated pathways is the presence of several proinflammatory signaling pathways whereas, in contrast to SAT, several metabolic pathways are downregulated including the general “metabolic pathways.” Together, these observations support the existing notion that SAT maintains, or even enhances, the metabolic phenotype while VAT loses metabolic capacity and shifts to a proinflammatory phenotype. These findings more broadly highlight the major impact of obesity on VAT, whereas SAT appears minimally affected and may even respond by upregulating lipid handling, potentially preserving AT function. Many of these findings from the KEGG pathway analysis are further supported by the GO analyses as they pertain to specific terms associated with BP and MF categories.

These findings are in line with past studies, including those comparing VAT and SAT (22–27). However, to our knowledge, this is the first study to evaluate DEGs in distinct AT with a focus on opposite expression patterns in response to a long-term high-fat diet and obesity. The existence of distinct metabolic obese phenotypes, i.e., healthy versus unhealthy wherein the latter is associated with accumulation of VAT (1–7), indicates a dichotomy in AT dysfunction driven by pathophysiological alterations specifically in VAT but may also denote adaptations in SAT that preserve AT function under obesogenic conditions. When identifying genes in SAT versus VAT that were oppositely regulated following a long-term high-fat diet, we were surprised to find only four genes that exhibited such a profile and that this was only modestly increased to 11 genes when reducing statistical stringency.

The two genes that were identified to be downregulated in SAT and upregulated in VAT were *trdn* and *hspb7*. The *trdn* gene, which encodes the protein triadin, is associated with Ca^2+^ handling in the sarcoplasmic reticulum of muscle cells through interactions with the ryanodine receptor (28, 29), although its role in AT is not defined. Similar to our observations in VAT of obese mice, the expression of *trdn* was also shown to be substantially upregulated in skeletal muscle of Caucasian women with obesity, however, a specific role for triadin was not investigated (30). Furthermore, a recent study showed that *TRDN* was upregulated in SAT of humans with morbid obesity and type II diabetes mellitus as compared with nondiabetic, body mass index (BMI)-matched individuals further implicating a role for triadin in disease progression in AT (31). In contrast, *Trdn* expression was shown to be significantly downregulated in brown adipose tissue (BAT) of obese rats relative to lean controls, highlighting potential AT-specific roles for triadin in health and disease (32). A speculative role for the upregulation of triadin may be in the form of contributing to increased cytosolic Ca^2+^, which could impact a variety of functions including promoting apoptosis and/or the production of proinflammatory adipokines, whereas its downregulation may afford protection against Ca^2+^ overload.

Heat shock protein b7 (HSPB7), encoded by *hspb7*, is a relatively understudied heat shock protein that has been shown to be protective against cardiomyopathies and cancer in preclinical studies (33–35). The upregulation of *hspb7* in VAT may be indicative of a stress response in obesity, however, the concomitant downregulation of this gene in SAT may underly an alternative role for this gene in AT that promotes AT dysfunction. Recent in vitro studies suggest a role for HSPB7 in inhibiting adipogenesis which, in the context of upregulated *hspb7* we observed in VAT, may contribute to AT dysfunction under obesogenic conditions and nutrient overload (36). An earlier study showed that *Hspb7* was upregulated in BAT and epididymal VAT of obese Zucker rats (37). Although we did not investigate BAT in the present study, the observation that *Hspb7* similarly increases in a distinct VAT depot using a different rodent model of obesity represents a compelling reason to understand the role of this gene in mediating AT dysfunction.

We also identified two genes with the opposite expression profile: both *elovl6* and *kcnj15* were upregulated in SAT and downregulated in VAT with obesity. The latter encodes a K^+^ ion channel, Kir4.2, that was most recently shown to play a pathological role in the kidney in response to low-dietary K^+^ (38). *KCNJ15* has also been identified as a susceptibility gene to type II diabetes mellitus though the focus to date has been on pancreatic β cells where Kir4.2 function is proposed to inhibit glucose-stimulated insulin secretion (39, 40). These findings would suggest a detrimental role for this gene in obesity perhaps via inducing insulin resistance, however, a role for this gene in AT is yet to be defined.

Similarly, *elovl6* encodes the enzyme elongation of long-chain fatty acids family member 6 (Elovl6), which has been linked to insulin resistance (41, 42). Such evidence has positioned Elovl6 as a candidate target in ameliorating insulin resistance through preventing dysregulated lipid metabolism (43), however, tissue-specific roles remain unclear and the use of global knockout mice may mask the role of *elovl6* in SAT. Indeed, while both genes that we show to be upregulated in SAT and downregulated in VAT have been associated with metabolic disease processes and seemingly support studies showing dysfunctional SAT with obesity (44–46), their AT-specific functions remain to be determined. Based on the differential AT expression patterns of these four genes (*trdn*, *hspb7*, *elovl6*, and *kcnj15*), we propose a deeper investigation with respect to distinct AT depots to determine if manipulating the expression/function of these genes may resolve VAT dysfunction in obesity.

Previous studies have used bulk and single-cell (sc)/nucleus RNA sequencing to evaluate AT gene expression profiles in humans and animal models under a variety of conditions. Most recently, Norreen-Thorsen et al. (47) showed using an integrative correlation analysis of bulk RNA sequencing data, a method aimed at better evaluating adipocyte genes to overcome technical challenges encountered when using scRNA sequencing methods, both sex and AT depot-specific differences using human datasets with males exhibiting a subset of unique, cell type-enriched genes. However, obesity was not a focus. Although we did not include female mice in the present study and were therefore unable to detect sex differences in AT gene expression, the aforementioned study highlights potential important differences as it pertains to the progression of conditions such as cardiovascular disease with which men are more prone earlier in life (48, 49). With regard to obesity, and in contrast to the present study, several studies evaluated SAT or VAT independently to determine obesity-induced gene alterations in AT (23, 27, 44–46). Although our findings support those studies investigating VAT depots in both human and rodent models of obesity (23, 27), several of the studies identified increased proinflammatory gene expression profiles in SAT of women and children with obesity as well as in a porcine model of obesity (44–46). These findings indicate that SAT may serve as an additional source of AT dysfunction under specific conditions or in different model systems, however, the absence of VAT data in these studies does not allow for a comparison in the degree of inflammation between the distinct AT depots. Furthermore, Massier et al. (24) recently integrated the work of several studies in a meta-analysis comparing SAT and VAT (omental and perivascular) and elegantly detailed novel cellular proportions, subtypes, and distinct clusters of progenitor cells between AT types ultimately highlighting cell-cell interactions and cellular relationships that may also dictate distinct alterations to AT in obesity. In summary, our present RNA-sequencing dataset offers a new outlook on SAT versus VAT in response to long-term obesity and nutrient overload and reveals genes that are differentially expressed in the two AT types. We propose that these genes in particular should be investigated further to determine their role in VAT dysfunction and/or putative SAT adaptive responses to poor nutrition and obesity.

### Limitations and Further Considerations

There are limitations to address in the present study. First, only male mice were used. Obesity is well-known to affect men and women differently, especially with regard to body fat distribution, adipocyte function, and proclivity to insulin resistance, and this could certainly impact gene expression across SAT and VAT depots (50, 51). Furthermore, although the lean controls were age-matched to the obese group in our study, the final ages of the mice at euthanasia were between 14.5 and 16.5 mo. Therefore, the cohort of mice used in this study can also be considered aged, however, a younger group of mice were not included to make such comparisons. A similar study design including a young group of mice is warranted to determine the impact of age, as well as a short-term diet regimen, on the AT transcriptome.

We also propose several considerations for the findings in the present study and future work. Here, we used a high-fat diet containing 42% kcal from fat, however, several groups have used a 60% kcal from fat diet as well as genetic models to induce obesity across numerous studies. It is likely that different diets, as well as time on a given diet, and distinct models of obesity would differentially impact AT genes from what we observed in this study. Future studies may build upon this work both from the perspective of specific genes identified and in magnitude of fold changes observed in ubiquitously influenced genes across diet types/regimens and models. Along these lines, it would be beneficial to provide a spectrum of obesity using a large cohort of mice that would allow for correlating gene expression profiles to specific physiological indicators (e.g., body weight, AT weights, blood glucose/lipids, etc.) as well as behavioral modifications that may be influenced by high-fat feeding and obesity (e.g., food intake, physical activity/sedentary behavior, etc.). Such analyses may improve our understanding of associated obesogenic variables and the relationship to changes in AT gene expression in response to obesity. Lastly, it should be considered that, in light of the drastic differences we observed in VAT versus SAT with regard to altered pathways and key nodes, targeting such affected pathways may provide ample opportunity for future research aimed at ameliorating AT dysfunction.

## DATA AVAILABILITY

Sample description, sequence, and gene expression data have been deposited in the NCBI under BioProject PRJNA1123180 with GEO series record GSE269663. Supplemental figures and tables can be found online at https://doi.org/10.6084/m9.figshare.26937643.

## SUPPLEMENTAL MATERIAL

10.6084/m9.figshare.26937643 Supplemental Tables S1–S3 and Supplemental Figs. S1 and S2: https://doi.org/10.6084/m9.figshare.26937643.

## GRANTS

This work was supported by an Institutional Development Award from the National Institute of General Medical Sciences of the National Institutes of Health under Award No. 2P20GM113125 (to I.S.F.). The Bioinformatics Data Science Core is supported by Delaware INBRE (Grant P20GM103446). M.A. is supported by the Saudi Arabian Cultural Mission.

## DISCLOSURES

No conflicts of interest, financial or otherwise, are declared by the authors.

## AUTHOR CONTRIBUTIONS

M.A. and I.S.F. conceived and designed research; M.A., M.S., and B.F.K. performed experiments; M.A., H.A., J.D.B., and S.W.P. analyzed data; M.A., H.A., M.S., J.D.B., B.F.K., S.W.P., and I.S.F. interpreted results of experiments; M.A., H.A., and I.S.F. prepared figures; M.A. and I.S.F. drafted manuscript; M.A., H.A., M.S., J.D.B., B.F.K., S.W.P., and I.S.F. edited and revised manuscript; M.A., H.A., M.S., J.D.B., B.F.K., S.W.P., and I.S.F. approved final version of manuscript.

## References

[1] Blüher M. Metabolically healthy obesity. Endocr Rev 41: bnaa004, 2020. doi:10.1210/endrev/bnaa004. 32128581 PMC7098708

[2] Cesaro A, De Michele G, Fimiani F, Acerbo V, Scherillo G, Signore G, Rotolo FP, Scialla F, Raucci G, Panico D, Gragnano F, Moscarella E, Scudiero O, Mennitti C, Calabrò P. Visceral adipose tissue and residual cardiovascular risk: a pathological link and new therapeutic options. Front Cardiovasc Med 10: 1187735, 2023. doi:10.3389/fcvm.2023.1187735. 37576108 PMC10421666

[3] Després JP. Body fat distribution and risk of cardiovascular disease: an update. Circulation 126: 1301–1313, 2012. doi:10.1161/CIRCULATIONAHA.111.067264. 22949540

[4] Gruzdeva O, Borodkina D, Uchasova E, Dyleva Y, Barbarash O. Localization of fat depots and cardiovascular risk. Lipids Health Dis 17: 218, 2018. doi:10.1186/s12944-018-0856-8. 30219068 PMC6138918

[5] Smith GI, Mittendorfer B, Klein S. Metabolically healthy obesity: facts and fantasies. J Clin Invest 129: 3978–3989, 2019. doi:10.1172/JCI129186. 31524630 PMC6763224

[6] Raheem J, Sliz E, Shin J, Holmes MV, Pike GB, Richer L, Gaudet D, Paus T, Pausova Z. Visceral adiposity is associated with metabolic profiles predictive of type 2 diabetes and myocardial infarction. Commun Med (Lond) 2: 81, 2022. doi:10.1038/s43856-022-00140-5. 35789567 PMC9249739

[7] Ruggiero AD, Vemuri R, DeStephanis D, Brock A, Block MR, Chou J, Das SK, Williams AG, Kavanagh K. Visceral adipose microbial and inflammatory signatures in metabolically healthy and unhealthy nonhuman primates. Obesity (Silver Spring) 31: 2543–2556, 2023. doi:10.1002/oby.23870. 37614163 PMC10783165

[8] Kalari KR, Nair AA, Bhavsar JD, O’Brien DR, Davila JI, Bockol MA, Nie J, Tang X, Baheti S, Doughty JB, Middha S, Sicotte H, Thompson AE, Asmann YW, Kocher JP. MAP-RSeq: mayo analysis pipeline for RNA sequencing. BMC Bioinformatics 15: 224, 2014. doi:10.1186/1471-2105-15-224. 24972667 PMC4228501

[9] Kim D, Langmead B, Salzberg SL. HISAT: a fast spliced aligner with low memory requirements. Nat Methods 12: 357–360, 2015. doi:10.1038/nmeth.3317. 25751142 PMC4655817

[10] Wang L, Wang S, Li W. RSeQC: quality control of RNA-seq experiments. Bioinformatics 28: 2184–2185, 2012. doi:10.1093/bioinformatics/bts356. 22743226

[11] Anders S, Pyl PT, Huber W. HTSeq–a Python framework to work with high-throughput sequencing data. Bioinformatics 31: 166–169, 2015. doi:10.1093/bioinformatics/btu638. 25260700 PMC4287950

[12] Robinson MD, McCarthy DJ, Smyth GK. edgeR: a Bioconductor package for differential expression analysis of digital gene expression data. Bioinformatics 26: 139–140, 2010. doi:10.1093/bioinformatics/btp616. 19910308 PMC2796818

[13] Kanehisa M, Furumichi M, Sato Y, Ishiguro-Watanabe M, Tanabe M. KEGG: integrating viruses and cellular organisms. Nucleic Acids Res 49: D545–D551, 2021. doi:10.1093/nar/gkaa970. 33125081 PMC7779016

[14] Kanehisa M, Furumichi M, Tanabe M, Sato Y, Morishima K. KEGG: new perspectives on genomes, pathways, diseases and drugs. Nucleic Acids Res 45: D353–D361, 2017. doi:10.1093/nar/gkw1092. 27899662 PMC5210567

[15] Sherman BT, Hao M, Qiu J, Jiao X, Baseler MW, Lane HC, Imamichi T, Chang W. DAVID: a web server for functional enrichment analysis and functional annotation of gene lists (2021 update). Nucleic Acids Res 50: W216–W221, 2022. doi:10.1093/nar/gkac194. 35325185 PMC9252805

[16] Bindea G, Mlecnik B, Hackl H, Charoentong P, Tosolini M, Kirilovsky A, Fridman WH, Pagès F, Trajanoski Z, Galon J. ClueGO: a Cytoscape plug-in to decipher functionally grouped gene ontology and pathway annotation networks. Bioinformatics 25: 1091–1093, 2009. doi:10.1093/bioinformatics/btp101. 19237447 PMC2666812

[17] Kawai T, Autieri MV, Scalia R. Adipose tissue inflammation and metabolic dysfunction in obesity. Am J Physiol Cell Physiol 320: C375–C391, 2021. doi:10.1152/ajpcell.00379.2020. 33356944 PMC8294624

[18] Altintas MM, Azad A, Nayer B, Contreras G, Zaias J, Faul C, Reiser J, Nayer A. Mast cells, macrophages, and crown-like structures distinguish subcutaneous from visceral fat in mice. J Lipid Res 52: 480–488, 2011. doi:10.1194/jlr.M011338. 21148461 PMC3035684

[19] Cancello R, Tordjman J, Poitou C, Guilhem G, Bouillot JL, Hugol D, Coussieu C, Basdevant A, Bar Hen A, Bedossa P, Guerre-Millo M, Clément K. Increased infiltration of macrophages in omental adipose tissue is associated with marked hepatic lesions in morbid human obesity. Diabetes 55: 1554–1561, 2006. doi:10.2337/db06-0133. 16731817

[20] Harman-Boehm I, Blüher M, Redel H, Sion-Vardy N, Ovadia S, Avinoach E, Shai I, Klöting N, Stumvoll M, Bashan N, Rudich A. Macrophage infiltration into omental versus subcutaneous fat across different populations: effect of regional adiposity and the comorbidities of obesity. J Clin Endocrinol Metab 92: 2240–2247, 2007. doi:10.1210/jc.2006-1811. 17374712

[21] O’Connell J, Lynch L, Cawood TJ, Kwasnik A, Nolan N, Geoghegan J, McCormick A, O’Farrelly C, O’Shea D. The relationship of omental and subcutaneous adipocyte size to metabolic disease in severe obesity. PLoS One 5: e9997, 2010. doi:10.1371/journal.pone.0009997. 20376319 PMC2848665

[22] Poulain-Godefroy O, Lecoeur C, Pattou F, Fruhbeck G, Froguel P. Inflammation is associated with a decrease of lipogenic factors in omental fat in women. Am J Physiol Regul Integr Comp Physiol 295: R1–R7, 2008. doi:10.1152/ajpregu.00926.2007. 18448614

[23] Qian Y, Sun H, Xiao H, Ma M, Xiao X, Qu Q. Microarray analysis of differentially expressed genes and their functions in omental visceral adipose tissues of pregnant women with vs. without gestational diabetes mellitus. Biomed Rep 6: 503–512, 2017. doi:10.3892/br.2017.878. 28529732 PMC5431681

[24] Massier L, Jalkanen J, Elmastas M, Zhong J, Wang T, Nono Nankam PA, Frendo-Cumbo S, Bäckdahl J, Subramanian N, Sekine T, Kerr AG, Tseng BTP, Laurencikiene J, Buggert M, Lourda M, Kublickiene K, Bhalla N, Andersson A, Valsesia A, Astrup A, Blaak EE, Ståhl PL, Viguerie N, Langin D, Wolfrum C, Blüher M, Rydén M, Mejhert N. An integrated single cell and spatial transcriptomic map of human white adipose tissue. Nat Commun 14: 1438, 2023. doi:10.1038/s41467-023-36983-2. 36922516 PMC10017705

[25] Konigorski S, Janke J, Patone G, Bergmann MM, Lippert C, Hübner N, Kaaks R, Boeing H, Pischon T. Identification of novel genes whose expression in adipose tissue affects body fat mass and distribution: an RNA-Seq and Mendelian Randomization study. Eur J Hum Genet 32: 1127–1135, 2024 [Erratum in *Eur J Hum Genet* 32: 1190, 2024]. doi:10.1038/s41431-022-01161-3. 35953519 PMC11369295

[26] Honecker J, Ruschke S, Seeliger C, Laber S, Strobel S, Pröll P, Nellaker C, Lindgren CM, Kulozik U, Ecker J, Karampinos DC, Claussnitzer M, Hauner H. Transcriptome and fatty-acid signatures of adipocyte hypertrophy and its non-invasive MR-based characterization in human adipose tissue. EBioMedicine 79: 104020, 2022. doi:10.1016/j.ebiom.2022.104020. 35490555 PMC9062743

[27] Sárvári AK, Van Hauwaert EL, Markussen LK, Gammelmark E, Marcher AB, Ebbesen MF, Nielsen R, Brewer JR, Madsen JGS, Mandrup S. Plasticity of epididymal adipose tissue in response to diet-induced obesity at single-nucleus resolution. Cell Metab 33: 437–453.e5, 2021. doi:10.1016/j.cmet.2020.12.004. 33378646

[28] Györke I, Hester N, Jones LR, Györke S. The role of calsequestrin, triadin, and junctin in conferring cardiac ryanodine receptor responsiveness to luminal calcium. Biophys J 86: 2121–2128, 2004. doi:10.1016/S0006-3495(04)74271-X. 15041652 PMC1304063

[29] Groh S, Marty I, Ottolia M, Prestipino G, Chapel A, Villaz M, Ronjat M. Functional interaction of the cytoplasmic domain of triadin with the skeletal ryanodine receptor. J Biol Chem 274: 12278–12283, 1999. doi:10.1074/jbc.274.18.12278. 10212196

[30] Paran CW, Verkerke AR, Heden TD, Park S, Zou K, Lawson HA, Song H, Turk J, Houmard JA, Funai K. Reduced efficiency of sarcolipin-dependent respiration in myocytes from humans with severe obesity. Obesity (Silver Spring) 23: 1440–1449, 2015. doi:10.1002/oby.21123. 25970801 PMC4483165

[31] Dong Z, Lei X, Kujawa SA, Bolu N, Zhao H, Wang C. Identification of core gene in obese type 2 diabetes patients using bioinformatics analysis. Adipocyte 10: 310–321, 2021. doi:10.1080/21623945.2021.1933297. 34085602 PMC8183531

[32] Joo JI, Yun JW. Gene expression profiling of adipose tissues in obesity susceptible and resistant rats under a high fat diet. Cell Physiol Biochem 27: 327–340, 2011. doi:10.1159/000327959. 21471722

[33] Mercer EJ, Lin YF, Cohen-Gould L, Evans T. Hspb7 is a cardioprotective chaperone facilitating sarcomeric proteostasis. Dev Biol 435: 41–55, 2018. doi:10.1016/j.ydbio.2018.01.005. 29331499 PMC5818303

[34] Naderi A. SRARP and HSPB7 are epigenetically regulated gene pairs that function as tumor suppressors and predict clinical outcome in malignancies. Mol Oncol 12: 724–755, 2018. doi:10.1002/1878-0261.12195. 29577611 PMC5928383

[35] Lin J, Deng Z, Tanikawa C, Shuin T, Miki T, Matsuda K, Nakamura Y. Downregulation of the tumor suppressor HSPB7, involved in the p53 pathway, in renal cell carcinoma by hypermethylation. Int J Oncol 44: 1490–1498, 2014. doi:10.3892/ijo.2014.2314. 24585183 PMC4027944

[36] Zhang S, van de Peppel J, Koedam M, van Leeuwen JPTM, van der Eerden BCJ. HSPB7 oppositely regulates human mesenchymal stromal cell-derived osteogenesis and adipogenesis. Stem Cell Res Ther 14: 126, 2023. doi:10.1186/s13287-023-03361-0. 37170285 PMC10173662

[37] Krief S, Faivre JF, Robert P, Le Douarin B, Brument-Larignon N, Lefrère I, Bouzyk MM, Anderson KM, Greller LD, Tobin FL, Souchet M, Bril A. Identification and characterization of cvHsp. A novel human small stress protein selectively expressed in cardiovascular and insulin-sensitive tissues. J Biol Chem 274: 36592–36600, 1999. doi:10.1074/jbc.274.51.36592. 10593960

[38] Terker AS, Zhang Y, Arroyo JP, Cao S, Wang S, Fan X, Denton JS, Zhang MZ, Harris RC. Kir4.2 mediates proximal potassium effects on glutaminase activity and kidney injury. Cell Rep 41: 111840, 2022. doi:10.1016/j.celrep.2022.111840. 36543132 PMC9827473

[39] Okamoto K, Iwasaki N, Doi K, Noiri E, Iwamoto Y, Uchigata Y, Fujita T, Tokunaga K. Inhibition of glucose-stimulated insulin secretion by KCNJ15, a newly identified susceptibility gene for type 2 diabetes. Diabetes 61: 1734–1741, 2012. doi:10.2337/db11-1201. 22566534 PMC3379671

[40] Okamoto K, Iwasaki N, Nishimura C, Doi K, Noiri E, Nakamura S, Takizawa M, Ogata M, Fujimaki R, Grarup N, Pisinger C, Borch-Johnsen K, Lauritzen T, Sandbaek A, Hansen T, Yasuda K, Osawa H, Nanjo K, Kadowaki T, Kasuga M, Pedersen O, Fujita T, Kamatani N, Iwamoto Y, Tokunaga K. Identification of KCNJ15 as a susceptibility gene in Asian patients with type 2 diabetes mellitus. Am J Hum Genet 86: 54–64, 2010. doi:10.1016/j.ajhg.2009.12.009. 20085713 PMC2801752

[41] Matsuzaka T, Shimano H, Yahagi N, Kato T, Atsumi A, Yamamoto T, Inoue N, Ishikawa M, Okada S, Ishigaki N, Iwasaki H, Iwasaki Y, Karasawa T, Kumadaki S, Matsui T, Sekiya M, Ohashi K, Hasty AH, Nakagawa Y, Takahashi A, Suzuki H, Yatoh S, Sone H, Toyoshima H, Osuga J, Yamada N. Crucial role of a long-chain fatty acid elongase, Elovl6, in obesity-induced insulin resistance. Nat Med 13: 1193–1202, 2007. doi:10.1038/nm1662. 17906635

[42] Zhao H, Matsuzaka T, Nakano Y, Motomura K, Tang N, Yokoo T, Okajima Y, Han SI, Takeuchi Y, Aita Y, Iwasaki H, Yatoh S, Suzuki H, Sekiya M, Yahagi N, Nakagawa Y, Sone H, Yamada N, Shimano H. Elovl6 deficiency improves glycemic control in diabetic *db/db* mice by expanding β-cell mass and increasing insulin secretory capacity. Diabetes 66: 1833–1846, 2017. doi:10.2337/db16-1277. 28461456

[43] Matsuzaka T. Role of fatty acid elongase Elovl6 in the regulation of energy metabolism and pathophysiological significance in diabetes. Diabetol Int 12: 68–73, 2020. doi:10.1007/s13340-020-00481-3. 33479581 PMC7790921

[44] Berardo C, Calcaterra V, Mauri A, Carelli S, Messa L, Destro F, Rey F, Cordaro E, Pelizzo G, Zuccotti G, Cereda C. Subcutaneous adipose tissue transcriptome highlights specific expression profiles in severe pediatric obesity: a pilot study. Cells 12: 1105, 2023. doi:10.3390/cells12081105. 37190014 PMC10137076

[45] Kogelman LJ, Cirera S, Zhernakova DV, Fredholm M, Franke L, Kadarmideen HN. Identification of co-expression gene networks, regulatory genes and pathways for obesity based on adipose tissue RNA sequencing in a porcine model. BMC Med Genomics 7: 57, 2014. doi:10.1186/1755-8794-7-57. 25270054 PMC4183073

[46] Messa L, Rey F, Pandini C, Barzaghini B, Micheletto G, Raimondi MT, Bertoli S, Cereda C, Zuccotti G, Cancello R, Carelli S. RNA-seq dataset of subcutaneous adipose tissue: transcriptional differences between obesity and healthy women. Data Brief 39: 107647, 2021. doi:10.1016/j.dib.2021.107647. 34901353 PMC8640228

[47] Norreen-Thorsen M, Struck EC, Öling S, Zwahlen M, Von Feilitzen K, Odeberg J, Lindskog C, Pontén F, Uhlén M, Dusart PJ, Butler LM. A human adipose tissue cell-type transcriptome atlas. Cell Rep 40: 111046, 2022. doi:10.1016/j.celrep.2022.111046. 35830816

[48] Bots SH, Peters SAE, Woodward M. Sex differences in coronary heart disease and stroke mortality: a global assessment of the effect of ageing between 1980 and 2010. BMJ Glob Health 2: e000298, 2017. doi:10.1136/bmjgh-2017-000298. 28589033 PMC5435266

[49] Stanhewicz AE, Wenner MM, Stachenfeld NS. Sex differences in endothelial function important to vascular health and overall cardiovascular disease risk across the lifespan. Am J Physiol Heart Circ Physiol 315: H1569–H1588, 2018. doi:10.1152/ajpheart.00396.2018. 30216121 PMC6734083

[50] Chang E, Varghese M, Singer K. Gender and sex differences in adipose tissue. Curr Diab Rep 18: 69, 2018. doi:10.1007/s11892-018-1031-3. 30058013 PMC6525964

[51] Arner P, Viguerie N, Massier L, Rydén M, Astrup A, Blaak E, Langin D, Andersson DP. Sex differences in adipose insulin resistance are linked to obesity, lipolysis and insulin receptor substrate 1. Int J Obes (Lond) 48: 934–940, 2024. doi:10.1038/s41366-024-01501-x. 38491191 PMC11217000

